# Breast cancer risk and imprinting methylation in blood

**DOI:** 10.1186/s13148-015-0125-x

**Published:** 2015-09-04

**Authors:** Kristina Harrison, Gwen Hoad, Paula Scott, Louise Simpson, Graham W. Horgan, Elizabeth Smyth, Steven D. Heys, Paul Haggarty

**Affiliations:** Division of Lifelong Health, Rowett Institute of Nutrition and Health, University of Aberdeen, Greenburn Road, Bucksburn, Aberdeen, UK; Division of Applied Medicine, School of Medicine and Dentistry, University of Aberdeen, University Medical Buildings, Foresterhill, Aberdeen, UK; Biomathematics and Statistics Scotland, Aberdeen, UK; Aberdeen Royal Infirmary, Ward 308, Foresterhill, Aberdeen, UK

**Keywords:** Imprinting, Methylation, Breast cancer, Invasive ductal carcinoma, Ductal carcinoma in situ

## Abstract

**Background:**

Altered DNA methylation of imprinted genes has been implicated in a range of cancers. Imprinting is established early in development, and some are maintained throughout the life course in multiple tissues, providing a plausible mechanism linking known early life factors to cancer risk. This study investigated methylation status of seven imprinted differentially methylated regions—*PLAGL1/ZAC1*, *H19*-ICR1, *IGF2-*DMR2, KvDMR-ICR2, *RB1*, *SNRPN-*DMR1 and *PEG3*—in blood samples from 189 women with the most common type of invasive breast cancer (invasive ductal carcinoma—IDC), 41 women with in situ breast cancer (ductal carcinoma in situ—DCIS) and 363 matched disease-free controls.

**Results:**

There was no evidence that imprinted gene methylation levels varied with age (between 25 and 87 years old), weight or height. Higher *PEG3* methylation was associated with an elevated risk of IDC (odds ratio (OR) 1.065; 95 % confidence interval (CI) 1.002, 1.132; *p* = 0.042) and DCIS (OR 1.139; 95 % CI 1.027, 1.263; *p* = 0.013). The effect was stronger when in situ and invasive breast cancer were combined (OR 1.079; 95 % CI 1.020, 1.142; *p* = 0.008). DCIS breast cancer risk increased with higher KvDMR-ICR2 methylation (OR 1.395; 95 % CI 1.190, 1.635; *p* < 0.001) and lower *PLAGL1/ZAC1* methylation (OR 0.905; 95 % CI 0.833, 0.982; *p* = 0.017). In a combined model, only KvDMR-ICR2 methylation remained significantly associated.

**Conclusions:**

These findings may help to improve our understanding of the aetiology of breast cancer and the importance of early life factors in particular. Imprinting methylation status also has the potential to contribute to the development of improved screening and treatment strategies for women with, or at risk of, breast cancer.

## Background

Breast cancer is the most common type of cancer in women in the UK, with a lifetime risk of one in eight [1]. Improvements in detection and treatment have increased survival rates, and currently, 85 % of women survive more than 5 years after diagnosis [1]. However, it is still the most common cause of cancer death in the world for women, with incidence markedly increasing [2]. The heritability of breast cancer is estimated at about 30 % but known genetic risk variants (e.g. *BRCA1*, *BRCA2*, *TP53* and *PTEN*) only account for around 5–10 % of all cases [3, 4]. Non-genetic factors such as obesity, alcohol consumption, diet, birth weight and exposure to oestrogen have all been linked to breast cancer risk, but the mechanisms underpinning these associations have yet to be identified [5, 6].

A common observation in human breast cancers, and many other tumour types, is epigenetic change, including altered methylation of DNA. There is an increasing interest in the role of epigenetics as a potential mechanism linking environmental exposures to cancer risk and as a non-genetic explanation for cancer heritability [7]. Aberrant DNA methylation has been reported in breast tumours for a number of genes involved in apoptosis, cell cycle control and DNA repair [8]. One class of epigenetic mark with particular relevance to cancer is imprinting; the epigenetic marking of genes in a parent-of-origin specific manner within the germ cells [9]. There is intra-individual variation of imprinting DNA methylation, and the early factors that influence this variability and the potential effects on later life cancer risk are of particular interest [10–12]. Once set, many germ line imprints are maintained in a wide range of adult somatic tissues across the life course [13, 14]. A number of imprinted genes are known tumour suppressors or oncogenes, and loss of imprinting is a hallmark of many cancers [8, 15, 16]. Interestingly, relaxation or loss of imprinting in apparently normal tissue of individuals with cancer or those at increased risk of the disease has been observed in normal colonic epithelium and normal breast tissue [17, 18]. These observations are relevant to the concept of field cancerisation—changes in normal tissue that predispose to the development of cancer—and the possible role of early factors in programming breast cancer susceptibility [10, 19]. The soma-wide nature of imprinting also makes this signal useful to consider as a potential biomarker of breast cancer.

DNA methylation changes in blood have been reported in breast cancer [20–22] and in relation to the pathological characteristics of breast cancer including histological type, tumour size and receptor status [23, 24]. Some apparent changes in blood DNA methylation in disease states could simply reflect changes in blood cell types [25, 26], and one advantage of studying imprinted genes is that they are unaffected by cellular heterogeneity [27].

The present study was undertaken to determine whether breast cancer risk was associated with altered DNA methylation of selected imprinted differentially methylated regions (DMRs) in non-tumour tissue (blood). Six germ line DMRs, identified to regulate the associated imprinted gene regions, were chosen for pyrosequencing analysis: *PLAGL1/ZAC1* [OMIM 603044], *H19* [OMIM 103280], KvDMR [OMIM 604115], *RB1* [OMIM 614041], *SNRPN* [OMIM 18227] and *PEG3* [OMIM 601483]. A somatic region (DMR2) linked with *IGF2* [OMIM 147470] was also selected for analysis. This DMR interacts with *H19* on the methylated paternal allele [28], has been linked with *IGF2* expression [29] and has been associated with nutritional in utero exposures [12, 30] and birth outcomes [31]. Methylation status was determined in blood samples collected at diagnosis and prior to treatment from women with the most common type of invasive breast cancer (invasive ductal carcinoma—IDC), the most common type of in situ cancer (ductal carcinoma in situ—DCIS) and disease-free controls. Subjects were matched for age, height, weight, body mass index (BMI) and menopausal status. A growing number of studies investigating tumour and non-tumour DNA methylation levels use array technologies such as the Illumina Infinium® HumanMethylation450 BeadChip [32] but this technology provides poor coverage of the imprinted regions of interest here. We investigated DNA methylation of these seven imprinted DMRs by pyrosequencing analysis.

## Results

The mean methylation levels for all imprinted DMRs are summarised in Table 1. These are in line with the predicted 50 % DNA methylation levels for imprinted genes, 100 % methylation on one allele and 0 % on the other. The mean *RB1* methylation was relatively high (68.61 %; SD = 4.65), but within the range of 33–70 % methylation, commonly reported for imprinted regions [33]. The somatic imprinted region *IGF2*-DMR2 had the highest inter-individual variability (48.50 %; SD = 5.72). There were no significant differences in age, height, weight and BMI between the matched cases and controls (Table 2).

**Table 1:** Average methylation levels for imprinted gene DMRs

Cohort	Imprinted gene DMRs						
	Chr. 6	Chr. 11			Chr. 13	Chr. 15	Chr. 19
	*PLAGL1*	*H19*	*IGF2*	KvDMR	*RB1*	*SNRPN*	*PEG3*
Combined	55.78 (4.92)	59.76 (4.19)	48.50 (5.72)	44.28 (2.36)	68.61 (4.65)	43.97 (3.00)	52.30 (2.99)
Disease-free	55.91 (4.92)	59.59 (4.35)	48.15 (5.77)	44.17 (2.34)	68.61 (4.30)	44.03 (3.03)	52.05 (2.93)
DCIS	53.90 (3.60)	59.70 (3.79)	49.52 (5.66)	45.81 (1.99)	68.22 (3.95)	43.62 (3.20)	53.29 (3.61)
IDC	55.92 (5.08)	60.10 (3.97)	48.94 (5.61)	44.13 (2.36)	68.68 (5.38)	43.93 (2.91)	52.57 (2.89)

Average percent methylation (%) reported (and standard deviation). Combined cohort included all disease-free women and breast cancer cases *n* = 593. Disease-free women *n* = 363. DCIS (ductal carcinoma in situ) *n* = 41. IDC (invasive ductal carcinoma) *n* = 189

*PLAGL1* pleomorphic adenoma gene like 1, *H19* H19, imprinted maternally expressed non-coding transcript-imprint control region 1, *IGF2* insulin-like growth factor 2*. KvDMR* 11p15 region imprint control region 2, *RB1* retinoblastoma 1. *SNRPN* small nuclear ribonucleoprotein polypeptide N, *PEG3* paternally expressed gene 3. DMR: differentially methylated region. Chr: chromosome

**Table 2:** Subject characteristics by disease status

Characteristic	Controls (*n* = 363)	Cases (*n* = 230)	*p* value
Age (years)	56 (11)	56 (11)	0.499
Height (m)	1.615 (0.07)	1.615 (0.06)	0.974
Weight (kg)	70.91 (14.25)	70.75 (13.50)	0.889
BMI (kg/m^2^)	27.20 (5.21)	27.14 (4.97)	0.902

Mean values with standard deviations (sd) shown in brackets. *p* values calculated using a two-sample Student’s *t* test

**Fig. 1:**
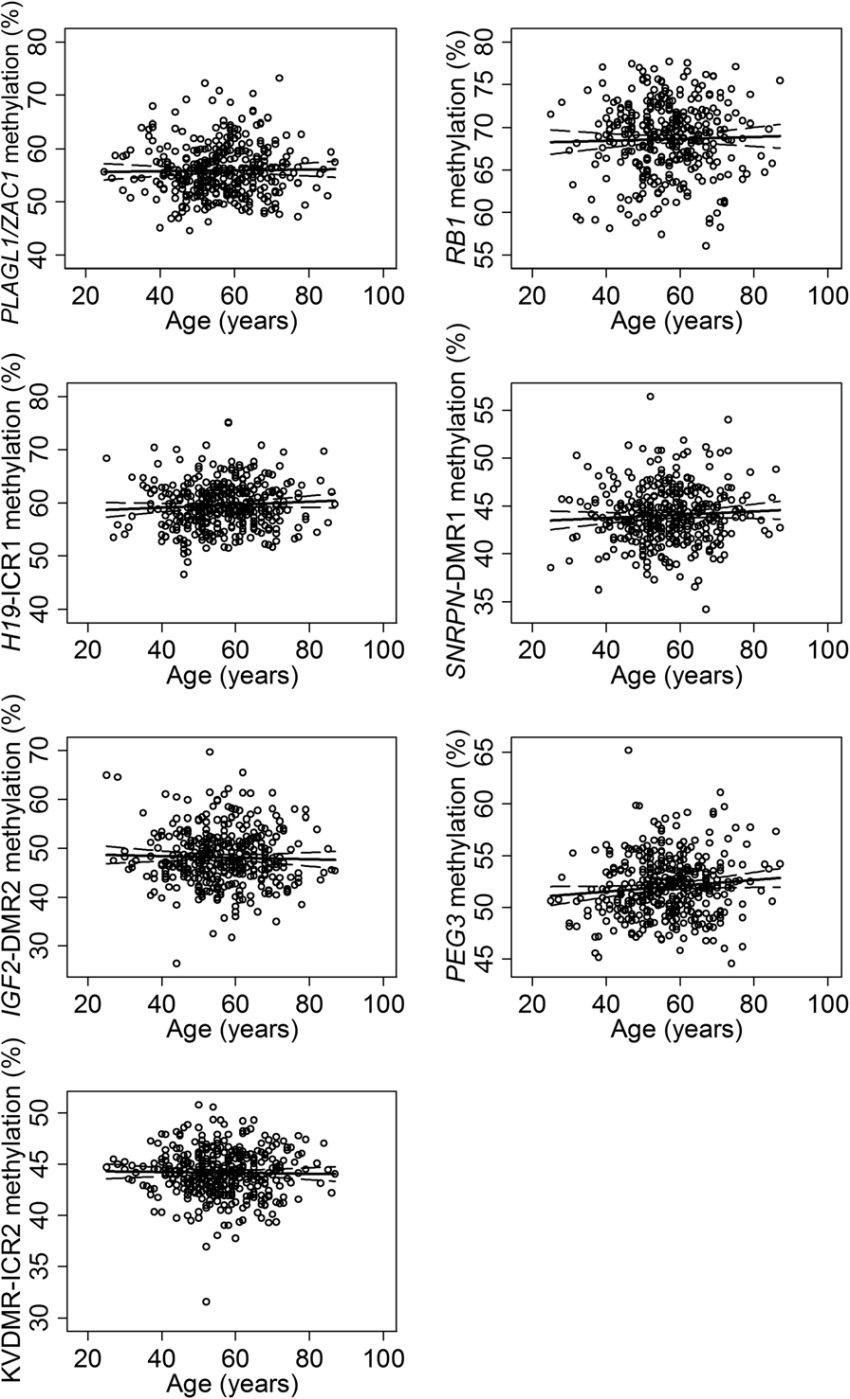
Imprinted gene methylation and age in disease-free women. Average methylation (%) within imprinted genes *PLAGL1/ZAC1*, *RB1*, *H19*-ICR1, *SNRPN*-DMR1, *IGF2*-DMR2, *PEG3* and KvDMR-ICR2 according to age for all disease-free samples. Linear regression analysis fit shown with a *solid line* and with 95 % CI shown with a *dashed line*. Average methylation levels are shown by an *open circle*. None of the associations were statistically significant. *PLAGL1/ZAC1* pleomorphic adenoma gene like 1, *RB1* retinoblastoma 1, *H19* H19, imprinted maternally expressed non-coding transcript-imprint control region 1, *SNRPN* small nuclear ribonucleoprotein polypeptide N, *IGF2* insulin-like growth factor 2*, PEG3* paternally expressed gene 3, *KvDMR* 11p15 region imprint control region 2, *CI* confidence intervals

The methylation level for the selected imprinted DMRs is shown for all of the disease-free women (Fig. 1). There was no evidence for a change in methylation of imprinted DMRs with age in this cross-sectional analysis. Neither were the imprinted DMR methylation levels significantly linked with weight or BMI of the disease-free women. Height was associated with *RB1* methylation, although this association was relatively weak (regression coefficient = 0.007; 95 % CI 0.000, 0.014; *p* = 0.049; *r*^2^ = 0.0116).

**Fig. 2:**
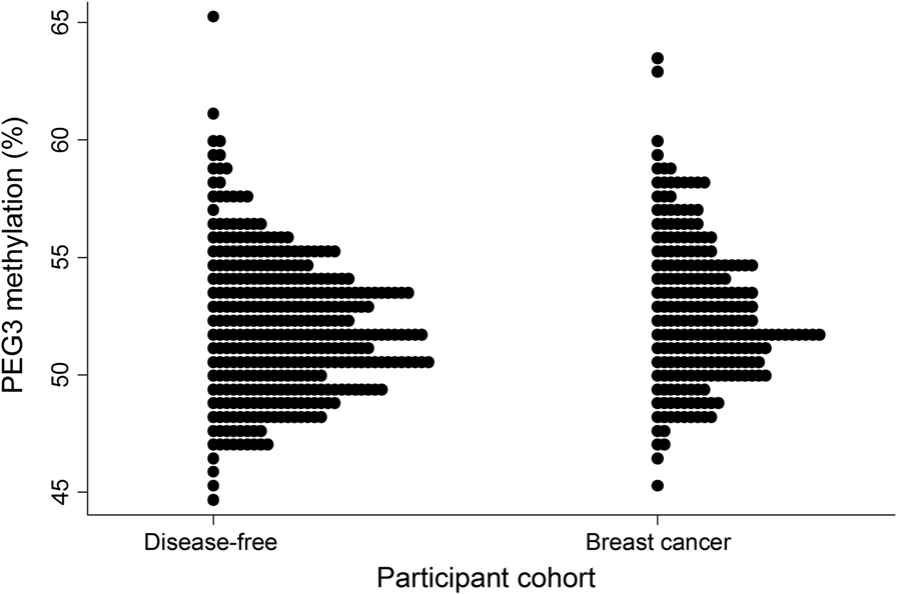
Distribution of average *PEG3* methylation levels. The distribution of the average *PEG3* methylation levels (percent—%) are presented for each participant cohort: disease-free women and breast cancer cases. Breast cancer cases include IDC and DCIS patients. Each participant is represented by a *filled circle. PEG3* paternally expressed gene 3, *IDC* invasive ductal carcinoma, *DCIS* ductal carcinoma in situ

Increased *PEG3* methylation was associated with an elevated risk of both IDC (odds ratio = 1.065; 95 % CI 1.002, 1.132; *p* = 0.042) and DCIS (odds ratio = 1.139; 95 % CI 1.027, 1.263; *p* = 0.013) compared to the controls (Table 3). This effect was more significant when DCIS and IDC were combined (odds ratio = 1.079; 95 % CI 1.020, 1.142; *p* = 0.008). There was no significant difference in *PEG3* methylation between the IDC and DCIS cases. The distribution of *PEG3* methylation according to disease status is shown in Fig. 2. *IGF2* methylation was also higher in combined IDC and DCIS cases compared to controls but this effect did not achieve statistical significance (*p* = 0.068).

**Table 3:** Logistic regression analysis of the effect of methylation level of imprinted gene DMRs on disease risk

Imprinted gene DMRs						
Chr. 6	Chr. 11			Chr. 13	Chr. 15	Chr. 19
*PLAGL1/ZAC1*	*H19-*ICR1	*IGF2-*DMR2	KvDMR-ICR2	*RB1*	*SNRPN-*DMR1	*PEG3*
All Cases vs. Controls						
0.987 [0.953,1.022] p = 0.462	1.026 [0.986,1.068] p = 0.210	1.028 [0.998,1.059] p = 0.068	1.049 [0.976,1.128] p = 0.193	1.001 [0.965,1.038] p = 0.947	0.985 [0.930,1.043] p = 0.601	1.079** [1.020,1.142] p = 0.008
IDC vs. Controls						
1.002 [0.966,1.039] p = 0.932	1.030 [0.987,1.074] p = 0.171	1.025 [0.994,1.057] p = 0.123	0.993 [0.920,1.072] p = 0.860	1.006 [0.967,1.046] p = 0.772	0.991 [0.932,1.054] p = 0.932	1.065* [1.002,1.132] p = 0.042
DCIS vs. Controls						
0.905* [0.833,0.982] p = 0.017	1.007 [0.932,1.088] p = 0.862	1.039 [0.984,1.099] p = 0.170	1.395*** [1.190,1.635] p < 0.001	0.976 [0.904,1.053] p = 0.530	0.954 [0.851,1.068] p = 0.412	1.139* [1.027,1.263] p = 0.013
DCIS vs. IDC						
0.904* [0.830,0.983] p = 0.018	0.967 [0.882,1.060] p = 0.474	1.021 [0.961,1.084] p = 0.509	1.413*** [1.168,1.681] p < 0.001	0.984 [0.924,1.048] p = 0.613	0.967 [0.856,1.092] p = 0.586	1.084 [0.971,1.270] p = 0.150

Logistic regression analysis, reporting odds ratios [95 % confidence intervals]. *p < 0.05, **p < 0.01, ***p < 0.001. Analysis adjusted for menopausal status, age and weight. All cases cohort describes combined IDC (invasive ductal carcinoma) and DCIS (ductal carcinoma *in-situ*) cases. DMR: differentially methylated region. Chr: chromosome. *PLAGL1/ZAC1:* Pleomorphic adenoma gene like 1. *H19*: H19, imprinted maternally expressed non-coding transcript-Imprint control region 1. *IGF2*: Insulin-like growth factor 2*.* KvDMR: 11p15 region imprint control region 2. *RB1*: Retinoblastoma 1. *SNRPN*: Small nuclear Ribonucleoprotein polypeptide N. *PEG3*: Paternally expressed gene 3

Two other DMRs exhibited differences in methylation in the DCIS cases compared to the controls (Table 3). Increased KvDMR-ICR2 methylation was associated with an elevated risk of DCIS (odds ratio = 1.395; 95 % CI 1.190, 1.635; *p* < 0.001). Increased KvDMR-ICR2 methylation was also observed for the DCIS cases compared to the IDC cases (Odds Ratio = 1.413; 95 % CI 1.168, 1.681; *p* < 0.001), confirming this association within DCIS cases only. Risk of DCIS decreased with increasing *PLAGL1/ZAC1* methylation (odds ratio = 0.905; 95 % CI 0.833, 0.982; *p* = 0.017) (Table 3). Again, a significant difference was observed between the DCIS and IDC cases (odds ratio = 0.904; 95 % CI 0.830, 0.983; *p* = 0.018), confirming the effects for *PLAGL1/ZAC1* methylation to be specific for the DCIS cases. Further statistical analysis showed that *PLAGL1/ZAC1* methylation was inversely correlated with KVDMR-ICR2 (correlation coefficient = −0.290; *p* < 0.001). When both *PLAGL1/ZAC1* and KVDMR-ICR2 were included in the same logistic regression model, only KvDMR-ICR2 remained significant (data not shown).

An overview of the pathological characteristics of IDC are summarised in Table 4. Further analysis based on subsets of the data produced some significant associations but the numbers in these comparisons were small and the results are included here primarily as hypothesis generating for future studies. Decreasing *SNRPN* methylation was associated with higher IDC histological grade (grade 3) compared to low histological grade 1 IDC (odds ratio = 0.808; 95 % CI 0.674, 0.969; *p* = 0.022) and combined histological grades 1 and 2 (odds ratio = 0.833; 95 % CI 0.711, 0.976; *p* = 0.024). Decreasing *SNRPN* methylation was also linked to increasing tumour size (regression coefficient = −0.047; 95 % CI −0.076, −0.019; *p* = 0.001). Both of these characteristics are associated with a poorer prognostic outcome. Higher *SNRPN* methylation was linked to a ‘good’ Nottingham Prognostic Index (NPI) prognostic category compared to a ‘moderate’, ‘poor’ or combined ‘moderate’ and ‘poor’ category (data not shown). The NPI category is partly determined by IDC grade and tumour size. Increased KvDMR-ICR2 methylation appeared to be associated with oestrogen receptor (ER) positive status, after adjustment for breast cancer type (odds ratio = 1.197; 95 % CI 1.032, 1.389; *p* = 0.018). No other pathological features (including progesterone receptor—PR or human epidermal growth factor 2—HER2 status) were significantly associated with methylation levels.

**Table 4:** Pathological characteristics of IDC cases, according to menopausal status

Characteristic	All cases (*n* = 189)	Pre-menopausal (*n* = 66)	Post-menopausal (*n* = 123)
Grade			
Grade 1	23 (12.2 %)	6 (9 %)	17 (13.8 %)
Grade 2	84 (44.4 %)	30 (45.5 %)	54 (43.9 %)
Grade 3	81 (42.9 %)	30 (45.5 %)	51 (41.5 %)
Missing info.	1 (0.5 %)	–	1 (0.8 %)
Lymph node involvement			
None	119 (63 %)	38 (57.6 %)	81 (65.9 %)
Axillary lymph nodes	69 (36.5 %)	28 (42.4 %)	41 (33.3 %)
Missing info.	1 (0.5 %)	–	1 (0.8 %)
Hormone receptor status			
ER positive	146 (77.2 %)	54 (81.8 %)	92 (74.8 %)
ER negative	43 (22.8 %)	12 (18.2 %)	31 (25.2 %)
PR positive	145 (76.7 %)	53 (80.3 %)	92 (74.8 %)
PR negative	44 (23.3 %)	13 (19.7 %)	31 (25.2 %)
HER2 receptor status			
Positive	22 (11.6 %)	11 (16.7 %)	11 (9 %)
Negative	164 (86.8 %)	54 (81.8 %)	110 (89.4 %)
Missing info.	3 (1.6 %)	1 (1.5 %)	2 (1.6 %)
Nottingham prognostic index (NPI)			
Good (NPI ≤ 3.4)	71 (37.6 %)	20 (30.3 %)	51 (41.5 %)
Moderate (NPI 3.41–5.4)	74 (39.1 %)	32 (48.5 %)	42 (34.2 %)
Poor (NPI > 5.41)	35 (18.5 %)	9 (13.6 %)	26 (21.1 %)
Missing info.	9 (4.8 %)	5 (7.6 %)	4 (3.2 %)

Participant numbers for pathological characteristics of IDC (invasive ductal carcinoma) patients overall and according to menopausal status (and percent—% of each characteristic)

*ER* oestrogen receptor, *PR* progesterone receptor, *HER2* human epidermal growth receptor 2

## Discussion

Cross-sectional analysis of all of the imprinted regions (*PLAGL1/ZAC1*, *H19*-ICR1, *IGF2-*DMR2, KvDMR-ICR2, *RB1*, *SNRPN-*DMR1 and *PEG3)* in the disease-free group indicated no change with age (between the age of 25 and 87 years). This reinforces the soma-wide nature of imprinting as blood cell type composition alters with age [34] which subsequently can influence methylation levels. These results support the view that imprinted regions are an attractive potential biomarker for examining breast cancer disease risk and progression, due to the stability of these throughout life [35].

Increased *PEG3* methylation was associated with an elevated risk of both IDC and DCIS compared to the controls, and the level of significance was greater when the two types of cancer were combined. The findings for *PEG3* in non-tumour tissue within this study mirror those previously reported in tumour samples: increased methylation of *PEG3* has been observed in tumour tissue of invasive breast and ovarian cancers [36], as well as invasive cervical cancer [37]. Studies have confirmed correlation of *PEG3* methylation between multiple tissue types, including blood and normal breast tissue [38] and that cancerous breast tumours have increased methylation compared to benign breast tumours [39]. *PEG3* encodes a Kruppel-type zinc-finger protein which regulates the tumour necrosis factor (TNF) response, of which dysregulation is commonly implicated in cancer [OMIM 601483]. Aberrant *PEG3* methylation in blood samples of women with breast cancer has recently been reported [39] but this analysis only highlighted the frequency of extreme outliers for *PEG3* methylation, rather than the more subtle changes in average methylation reported here. The same study also reported differential outlier frequency of *IGF2*-DMR2 in blood samples [39]. Average *IGF2-*DMR2 methylation was also increased in combined cases compared to controls in the study described here, but this effect only approached statistical significance. There are also reports of altered *IGF2* methylation in breast tumours [40].

Cancer marker studies suggest a common origin and development pathway of the two main types of breast cancer, IDC and DCIS. Reports have identified that low-grade DCIS can transition to low-grade IDC and high-grade DCIS to high-grade IDC [41, 42]. The fact that *PEG3* methylation in blood is associated with both DCIS and IDC separately in this study suggests that this change could contribute to the common origin and development pathway. This information could potentially contribute to the development of early biomarkers of breast cancer susceptibility.

Not all women with DCIS will go on to develop invasive breast cancer. The risk of progression is estimated at 25–50 % for women with low-grade DCIS and 75 % for women with high-grade DCIS [43]. A recent review suggested that screening asymptomatic women by mammography resulted in a 20 % reduction in mortality, but the authors also concluded that around 19 % of diagnosed cancers would not have caused a problem if left untreated [44] and others have suggested that there may be no survival advantage by breast screening, with the treatment itself being associated with a significant net morbidity and mortality [2].

An important clinical challenge is to identify which individuals can safely be left without treatment [45]. This study identified DNA methylation changes in blood samples associated with DCIS only. Increased KvDMR-ICR2 methylation and decreased *PLAGL1/ZAC1* methylation were linked with increasing risk of DCIS compared to both controls and IDC cases. When both genes were included in the same statistical model, only the significance for KvDMR-ICR2 methylation remained. Such findings have the potential to identify individuals with DCIS who are less likely to progress to invasive breast cancer. KvDMR-ICR2 regulates the *CDKN1C/KCNQ1* domain, including the non-coding antisense *KCNQ1OT1* RNA which silences genes on the paternal allele within this region. Downregulation of *CDKN1C* (which encodes p57(KIP2), a negative regulator of cell proliferation—OMIM 600856) has been associated with increased metastasis potential and poorer survival for breast cancer [46, 47]. Decreased *KCNQ1OT1* methylation has been identified in tumour development and the imprinting disorder Beckwith-Wiedemann syndrome which is associated with tumour predisposition [48]. Altered KvDMR-ICR2 methylation, as observed within this study, may influence breast cancer risk through an effect on *CDKN1C* or *KCNQ1OT1.* Further work on the links between KvDMR-ICR2 methylation and *CDKN1C/KCNQ1OT1* gene expression in cancer would be instructive.

Links between imprint signals in normal tissue (such as blood) and breast cancer risk are particularly relevant to the concept of ‘field cancerisation’ [19]. This is based on the idea that widespread changes in normal tissues precede the development of the disease and increase the risk of transition to cancer. There is a renewed interest in this paradigm in relation to epigenetic change, and it is particularly relevant to imprinting as many germ line DMRs stably maintain their allele specific methylation signal in a wide range of adult somatic tissues over the decades [38, 49, 50]. The soma-wide nature of certain imprinting signals, such as those studied here, makes them particularly convenient as biomarkers of breast cancer risk, type and prognosis since the signal should be detectable in easily accessible tissues such as blood. Cell-free circulating DNA (cfDNA) has also been used in epigenetic studies but these applications tend to be used for metastatic breast cancer [51]. The correlation between some imprinting methylation in tumour and normal tissue means that cfDNA originating from tumours could also potentially be used for this type of work but there is an extensive epigenetic change in tumours, and cfDNA may not always be relevant to investigations of cancer-predisposing epigenetic change in normal tissues.

The differences in imprinting methylation described here are also relevant to observations linking breast cancer susceptibility to early development [10]. A number of studies and meta-analyses have demonstrated a clear relationship between birth weight and breast cancer risk, though there is a debate as to the relative strength of the association for pre- and post-menopausal breast cancer [52–56]. The mechanism linking birth weight to breast cancer risk has not yet been established but imprinting could provide a plausible link, as one of the key functions of imprinted genes is to regulate foetal growth [57]. Imprinting methylation has also been linked to birth weight and health of the new born [31].

Interestingly, there is some evidence that methylation of imprinted genes may be modifiable by the early environment such as maternal nutrition before birth. One example of this is folic acid. This nutrient is metabolised through the pathway that provides methyl groups for DNA methylation, and its use in human pregnancy has been reported to influence *PEG3* and *IGF2* methylation in the offspring [12]. The methylation differences observed here are relatively small in magnitude, and this is generally the case in population epigenetic studies linking average methylation levels to phenotypes [10, 31, 58]. Human epigenetic studies almost universally measure the average methylation level in a sample of cells, usually blood or buccal cells, but the distribution and variance of methylation states may be more important [58]. Many human diseases have the potential to develop from a subset of cells, or even one cell as in cancer, and small changes in the average methylation level could reflect more important changes in the proportion of cells in high-risk epigenotype states [56]. From this study, we are not able to suggest a clear cut-off threshold to identify those individuals who will definitely develop breast cancer, but the measurement denotes statistical probability. We suggest that the significant regions are worth looking at in more depth to develop a more detailed picture of the methylation signal using methods such as next-generation bisulphite sequencing and that they may be used in conjunction with other risk factors to improve the overall predictive value of these imprinted gene regions.

## Conclusions

Altered DNA methylation has been shown to occur in tumours, with changes now being identified in non-tumour tissue such as blood samples. Imprinted gene methylation is particularly interesting to consider as these provide potential mechanistic routes to early life influences and non-genetic heritability of breast cancer. Using a case-control cohort of almost 600 women, we have observed increased *PEG3* methylation within blood samples of both invasive and in situ breast cancer cases. Increased KvDMR-ICR2 methylation was observed in women with in situ breast cancer compared to both controls and invasive breast cancers. DNA methylation stability was confirmed for the seven selected imprinted gene regions within this cohort. The findings set out in this study suggest potential biomarkers for breast cancer risk.

## Methods

### Study population

The study was approved by the North of Scotland Research Ethics Committee (reference number 08/S0801/17), and all participants gave informed consent. Two thousand and one hundred seventy-two women were recruited between 2008 and 2013 from the Aberdeen Breast Clinic with 1168 breast cancer cases and 1004 disease-free controls. The breast cancer patients were newly diagnosed, with histological confirmation and without detectable metastatic disease. Blood samples were taken prior to surgical intervention. The disease-free women had attended the Aberdeen Breast Clinic with benign breast changes, none of which significantly increased their risk of breast cancer. Disease-free status was confirmed by clinical examination, mammography, breast ultrasonography and fine needle aspiration cytology where appropriate.

Only European Caucasians were included in the study and women with known family history of *BRCA1/2* mutations and those which have been identified at an increased risk of breast cancer were excluded from the analysis. The women were identified as having an increased risk of breast cancer if there was known family history, previous occurrence of atypical ductal hyperplasia, lobular carcinoma in situ or Hodgkin’s disease.

Participant characteristics and pathological information including histological type, tumour size, tumour grade, lymph node status, hormone receptor status and HER2 expression status were recorded. Nottingham Prognostic Index was determined using tumour grade, tumour size and involvement of lymph nodes [59]. In the initial phase of the study, we included all types of cancer in the case group and matched an equivalent numbers of controls. After applying the study exclusion criteria and removing the more unusual cancer types (numbers too small for meaningful comparison), the number of controls (*n* = 363) exceeded the number of cases (*n* = 230) but the groups remained well matched for the key variables of age, height, weight, body mass index (BMI) and menopausal status.

### Participant characteristics

Age, height, weight and BMI of the participants are summarised in Table 2. The DCIS cases were classified as intermediate (27 %), intermediate/high (17 %) or high (56 %) grade cancers. Information on ER status was available for 78 % of the DCIS cases (of these, 84 % were positive and 16 % negative) and PR status for 76 % of the DCIS cases (of these, 74 % were positive and 26 % negative). Pathological characteristics for the IDC cases are summarised in Table 4.

### DNA methylation analysis

DNA was extracted from the blood using QIAamp DNA Mini Blood QIAcube kits (Qiagen, Crawley, UK), automated on a QIAcube (Qiagen, Crawley, UK). The DNA was quantified with SYBR® Green on a Rotergene Q (Qiagen, Crawley, UK) using DNA standards (Life Technologies, Paisley, UK). The DNA samples were dispensed into 96-well plates by a QIAgility robotic system (Qiagen, Crawley, UK). Matched samples (breast cancer cases and disease-free controls) were positioned on the same plate to minimise any potential batch effects. The samples were treated with sodium bisulphite using Epitect® 96 Bisulfite kits (Qiagen, Crawley, UK). Published PCR primers designed for bisulphite-converted DNA were used for *PLAGL1/ZAC1*, *IGF2-*DMR2, *SNRPN-*DMR1 and *PEG3* assays [60–63]. FASTA sequences were used to design specific assays using PyroMark Assay Design Software (version 2.0, Qiagen, Crawley, UK) for the *H19*-ICR1, KvDMR-ICR2 and *RB1* assays [33]. The primers were as follows: forward 5′-TGGGGATTTTGATGGGGTTA-3′, reverse 5′-biotin-CCTACTCCAAACATTATAAAAAAAACTAAC-3′ and sequencing primer 5′-GATGGTTAGGGTGTGTT-3′ for the *H19*-ICR1 assay; forward 5′-biotin-GGGTGATTATTGGAGTTGTTGAGGTGAG-3′, reverse 5′-TCCAATCCCAATTCAACCCACTC-3′ and sequencing primer 5′-CTAAACCACCATAAAAACTAT-3′ for the KvDMR-ICR2 assay; forward 5′-biotin-TGGGGTTAGGAGGTGAAAGTGG-3′, reverse 5′-CATATAAAACAACAACAAATCCCTTTCTAC-3′ and sequencing primer 5′-CCCTAAACCTACCTTCCC-3′ for the *RB1* assay. Pyrosequencing was performed using the PyroMark MD Q96 Instrument (Pyrosequencing, Inc., Uppsala, Sweden) running PyroMark CpG software (version 1.0.9, Biotage GB Ltd., Hengoed, UK). The plates were prepared for pyrosequencing according to the manufacturers’ protocol. Only data which passed appropriate quality control thresholds were included for analysis.

### Statistical analysis

Data were analysed using STATA/SE version 13 (Stata Corp, College Station, Texas, USA). Subject characteristics of the entire cohort and pathological characteristics of breast cancer cases were compared using Student’s *t* test. Pairwise correlation was run to determine the relationship between CpG sites within each gene/region. CpG sites within each gene (*PEG3* and *RB1*; seven CpG sites *H19-ICR1*, KvDMR-ICR2 and *PLAGL1*; six CpG sites *SNRPN* and *IGF2*; four CpG sites each) were highly correlated with each other (all at *p* < 0.01), and the average methylation for each of these genes/regions was used in subsequent analysis. Linear regression was used to determine the relationship between methylation and age, weight, height and body mass index in the disease-free control group.

Logistic regression was used to compare methylation levels in the cases (IDC only, DCIS only and IDC and DCIS combined) relative to the disease-free controls. IDC was also compared with DCIS. Logistic regression was used for other categorical outcomes (lymph node involvement and receptor status). Multinomial logistic regression was used to evaluate the link between methylation and NPI categories. All analyses were adjusted for appropriate covariates including menopausal status, age and weight (as stated in the tables and text). The results of regression analysis are presented with 95 % confidence intervals and *p* values. Significance was set at *p < 0.05, **p < 0.01 and ***p < 0.001. Due to the severe effect of multiple comparison adjustments on false negative error rates and the exploratory nature of this research, such adjustments have been avoided to prevent this.
